# Growth Hormone (GH) Hypersecretion and GH Receptor Resistance in Streptozotocin Diabetic Mice in Response to a GH Secretagogue

**DOI:** 10.1155/EDR.2003.73

**Published:** 2003

**Authors:** Peter B. Johansen, Yael Segev, Daniel Landau, Moshe Phillip, Allan Flyvbjerg

**Affiliations:** 1 Pharmacological Research 3 Novo Nordisk A/S Novo Nordisk Park Måløv DK-2760 Denmark; 2 Molecular Endocrinology Laboratory Department of Pediatrics Soroka University Medical Center Beer-Sheva Israel; 3 Medical Department M/Medical Research Laboratory Institute of Experimental Clinical Research Aarhus Kommunehospital Aarhus Denmark

## Abstract

The growth hormone (GH) and insulin-like growth factor I (IGF-I) axis were studied in streptozotocin (STZ) diabetic and nondiabetic female mice following intravenous (IV) injection of the GH secretagogue (GHS) ipamorelin or saline. On day 14, blood samples were obtained before and 10 minutes after the injection. Livers were removed and frozen for determination of the mRNA expressions of the GH receptor, GH-binding protein, and IGF-I, and hepatic IGF-I peptide. Serum samples were analyzed for GH and IGF-I. Following ipamorelin injection, the GH levels were found to be 150 ± 35 *μ*g/L and 62 ± 11 *μ*g/L in the diabetic compared to the nondiabetic mice (*P* < .05). Serum IGF-I levels were lower in diabetic than in nondiabetic animals, and rose after stimulation only in the nondiabetic animals. Furthermore, hepatic GH resistance and IGF-I mRNA levels and IGF-I peptide were increased in nondiabetic animals in response to GH stimulation, whereas the low levels per se of all these parameters in diabetic mice were unaffected. The study shows that STZ diabetic mice demonstrate a substantial part of the clinical features of type 1 diabetes in humans, including GH hypersecretion and GH resistance. Accordingly, it is proposed that STZ diabetic mice may be a better model of the perturbations of the GH/IGF-I axis in diabetes than STZ diabetic rats.


*Experimental Diab. Res.*, 4:73–81, 2003
Copyright © Taylor and Francis Inc.
ISSN: 1543-8600 print / 1543-8619 online
DOI: 10.1080/15438600390220016

### Growth Hormone (GH) Hypersecretion and GH Receptor Resistance in Streptozotocin Diabetic Mice in Response to a GH Secretagogue

**Peter B. Johansen,[1] Yael Segev,[2] Daniel Landau,[2] Moshe Phillip,[2] and Allan Flyvbjerg[3]**

[1]*Pharmacological Research 3, Novo Nordisk A/S, Måløv, Denmark*
[2]*Molecular Endocrinology Laboratory, Department of Pediatrics, Soroka University Medical Center, Beer-Sheva, Israel*
[3]*Medical Department M/Medical Research Laboratory, Institute of Experimental Clinical Research, Aarhus Kommunehospital, Aarhus, Denmark*

**The growth hormone (GH) and insulin-like growth factor I (IGF-I) axis were studied in streptozotocin (STZ) diabetic and nondiabetic female mice following intravenous (IV) injection of the GH secretagogue (GHS) ipamorelin or saline. On day 14, blood samples were obtained before and 10 minutes after the injection. Livers were removed and frozen for determination of the mRNA expressions of the GH receptor, GH-binding protein, and IGF-I, and hepatic IGF-I peptide. Serum samples were analyzed for GH and IGF-I. Following ipamorelin injection, the GH levels were found to be 150 ± 35 $\mu$g/L and 62 ± 11 $\mu$g/L in the diabetic compared to the nondiabetic mice ($P < .05$). Serum IGF-I levels were lower in diabetic than in nondiabetic animals, and rose after stimulation only in the nondiabetic animals. Furthermore, hepatic GH resistance and IGF-I mRNA levels and IGF-I peptide were increased in nondiabetic animals in response to GH stimulation, whereas the low levels per se of all these parameters in diabetic mice were unaffected. The study shows that STZ diabetic mice demonstrate a substantial part of the clinical features of type 1 diabetes in humans, including GH hypersecretion and GH resistance. Accordingly, it is proposed that STZ diabetic mice may be a better model of the perturbations of the GH/IGF-I axis in diabetes than STZ diabetic rats.**

**Keywords** Diabetes; Growth Hormone Resistance; Growth Hormone Secretion; Insulin-Like Growth Factors; Ipamorelin; Mice; Streptozotocin

Growth hormone (GH) and insulin-like growth factors (IGFs) have a long history in diabetes mellitus, with conceivable effects on the development of diabetic renal complications; for review, see [1–4]. Poorly controlled type 1 diabetes in man is characterized by GH hypersecretion and low circulating IGF-I levels [5–8]. The streptozotocin (STZ) diabetic rat, however, is characterized by suppressed serum GH levels and loss of the characteristic pulsatility [9–11]. It has been reported that pituitary GH concentration is significantly reduced in STZ diabetic rats as compared to that observed in control animals. The reduced GH levels are probably due to a deficiency in GH storage and/or synthesis [12]. Both increased [13] and decreased [14] GH levels have been found in the Biobred (BB) rat, which is considered to be the classical model of early (autoimmune) onset type 1 diabetes.

The highly conspicuous species difference in neuroregulation of the GH axis in diabetes is also present during other

Received 31 July 2002; accepted 24 February 2003.

This study was supported by grants from the Danish Medical Research Council (no. 9700592), the Novo Foundation, the Aage Louis-Hansen Memorial Foundation, the Nordic Insulin Foundation, the Eva and Henry Frænkels Memorial Foundation, and the Aarhus University-Novo Nordisk Center for Research in Growth and Regeneration (no. 9600822). The authors are grateful to Mrs. Karen Mathiassen, Kirsten Nyborg, and Ninna Rosenqvist for excellent technical assistance.

Address correspondence to Peter B. Johansen, PhD, Pharmacological Research 3, Novo Nordisk A/S, Novo Nordisk Park, DK-2760 Måløv, Denmark. E-mail: pjoh@novonordisk.com exogenous stimuli, such as exercise, starvation, and stress; for review, see [15]. To be able to extent results obtained in animal studies into humans, it is imperative that the animal model closely reflects the clinical condition. In the present paper, we show enhancement of GH secretion also in STZ diabetic mice following intravenous (IV) injection of the GH secretagogue (GHS) ipamorelin.

### MATERIALS AND METHODS

### Animals and Protocol

Four groups of adult female Balb/C(a) mice (Bomholtgaard, Ry, Denmark), with initial body weights of 15 to 19 g, were studied. The mice were housed 6 to 8 per cage in a room with 12:12-hour light cycle, temperature 21°C ± 2°C and humidity 55% ± 2%. The animals had free access to standard chow (Altromin no. 1324, Lage, Germany) and tap water throughout the experiment. The animals were randomly allocated to 4 groups, and 2 of these groups were made diabetic by a single IV injection of STZ (Upjohn Company, Kalamazoo, MI, USA), in a dose of 300 mg/kg body weight. The development of diabetes was followed over the next 14 days by measuring blood glucose by Haemoglucotest 1-44 and Reflolux II reflectance meter (Boehringer-Mannheim, Mannheim, Germany) and urine for glucose and ketone bodies by Neostix-4 (Ames, Stoke Poges, Slough, UK). Only animals with blood glucose above 16 mmol/L, glucosuria above 111 mmol/L, and without ketonuria were included in the study. Body weight and food consumption were recorded.

At day 14, ipamorelin, 200 $\mu$g/kg, or saline, 5 mL/kg, were injected intravenously. Blood was drawn before and exactly 10 minutes after the injection from the retro-orbital venous plexus for determination of serum GH, IGF-I, and IGF-binding protein 3 (IGFBP-3). Serum was stored at −80°C until measurements were performed. Furthermore, the kidneys and livers were rapidly removed and carefully cleaned and weighed. The livers were frozen in liquid nitrogen and stored at −80°C for later determination of the mRNA of the GH receptor (GHR), GH-binding protein (GHBP), and IGF-I, and hepatic IGF-I peptide.

The study was performed under an approved animal permit complying with national regulations for use of animals in research (the Danish Animal Inspectorate).

### Immunoassays

Serum GH was measured by radioimmunoassay (RIA) using a specific polyclonal rabbit rat(r) GH antibody and rGH as standard. Semilog linearity of mouse serum and rGH (in the standard) was found at multiple dilutions, indicating antigen similarity between mouse GH and rGH. The ingredients, including [$^{125}$I]-rGH, were obtained from Amersham International, Bucks, UK. Serum IGF-I was measured after extraction with acid-ethanol. The mixture was incubated for 2 hours at room temperature, centrifuged, and 25 $\mu$L of the supernatant was diluted 1:200 before analysis. Liver extraction was performed as previously described [16]. Briefly, 80 to 100 mg of liver tissue was homogenized on ice in 1 M acetic acid (5 mL/g tissue) with an Ultra Turrax TD 25 and further disrupted using a Potter Elvehjelm homogenizer. An additional extraction procedure (with ethanol/HCl) was performed in liver homogenates to fully remove all IGFBPs. After lyophilization, the samples were redissolved in phosphate buffer (pH 8.0) and kept at −80°C until the IGF-I assay was performed in diluted extracts. Serum and liver IGF-I levels were measured by RIA using a polyclonal rabbit antibody (Nichols Institute Diagnostics, San Capistrano, CA, USA) and recombinant human IGF-I as standard (Amersham International). The tissue IGF-I concentrations were corrected for the contribution of entrapped serum IGF-I [16]. Monoiodinated IGF-I ([$^{125}$I-Tyr$^{31}$]-IGF-I) was obtained from Novo Nordisk A/S, Bagsværd, Denmark. Intra- and interassay coefficients of variations for both assays were <5% and <10%, respectively, for both assays.

### Western Ligand Blotting (WLB) for Determination of Serum IGFBP-3

Sodium dodecyl sulfate–polyacrylamide gel electrophoresis (SDS-PAGE) and WLB were performed according to the method of Hossenlopp and colleagues [17] as previously described [18]. Two microliters of serum was subjected to SDS-PAGE (10% polyacrylamide) under nonreducing conditions. The electrophoresed proteins were transferred by electroelution onto nitrocellulose paper (Schleicher & Schuell, Munich, Germany) and membranes were incubated overnight at 4°C with approximately 500,000 cpm [$^{125}$I]-IGF-I (specific activity 2000 Ci/mmol) in 10 mL 10 mmol/L Tris-HCl buffer (TBS) containing 1% bovine serum albumin (BSA) and 0.1% Tween (pH 7.4). Membranes were washed with TBS and after drying overnight, the nitrocellulose sheets were autoradiographed with Kodak X-AR film and exposed to Du Pont–New England Nuclear enhancing screens at −80°C for 3 to 7 days. Specificity of the IGFBP bands was ensured by competitive coincubation with unlabeled IGF-I purchased from Bachem (Bubendorf, Switzerland). On WLB (with [$^{125}$I]-IGF-I as ligand), IGFBP-3 appear as a 38- to 42-kDa doublet band corresponding to the intact acid-stable IGF-binding subunit of IGFBP-3. Western ligand blots were quantified by densitometry using a Shimadzu CS-9001 PC dual-wavelength flying spot scanner.

### GHR/GHBP and IGF-I mRNA

Total RNA was prepared from frozen tissue by Tri reagent (Molecular Research Center, Cincinnati, OH, USA) and

**TABLE 1**
Body weight, blood glucose, food consumption, kidney weight (both kidneys), and liver weight in nondiabetic mice injected with saline (C/SAL) or ipamorelin (C/IPA) and diabetic animals injected with saline (D/SAL) or ipamorelin (D/IPA) at day 14

| | Body weight (g) | Blood glucose (mM) | Food consumption (g/day) | Kidney weight (mg) | Liver weight (mg) |
|---|---|---|---|---|---|
| C/SAL | 20.2 ± 0.4 | 6.1 ± 0.4 | 5.4 ± 0.9 | 245 ± 3 | 1178 ± 46 |
| C/IPA | 19.9 ± 0.3 | 5.9 ± 0.5 | 5.9 ± 0.5 | 244 ± 6 | 1074 ± 51 |
| D/SAL | 15.2 ± 0.3* | 22.8 ± 0.7* | 9.2 ± 0.8* | 269 ± 6* | 898 ± 38* |
| D/IPA | 15.5 ± 0.4* | 23.8 ± 1.0* | 9.6 ± 0.9* | 264 ± 6* | 902 ± 76* |

*Note*. Values are means ± SEM, n = 7–10 per group.
*$P < .01$ versus respective control groups (Student's $t$ test for unpaired comparisons).

quantified by absorbency at 260 nm. The integrity of the RNA was assessed by visual inspection of the ethidium bromide–stained 28S and 18S RNA bands after electrophoresis through 1.25%/2.2 M formaldehyde gels. For Northern blot analysis, 20 $\mu$g of total RNA were electrophoresed on 1.3% agarose/2.2 M formaldehyde gels in 3-morpholinopropanesulphonic acid buffer. The RNA was then transferred onto Magna-Graph (MSI, Westboro, MA, USA) nylon membranes and was cross-linked to the membrane with an ultraviolet (UV) cross-linker (Hoefer Scientific Instruments, San Francisco, CA, USA). A rat GHR/GHBP probe (a generous gift from L. Mathews) was radiolabeled with [$^{32}$P]-dCTP (Amersham International) by a random-primed DNA labeling kit (Boehringer-Mannheim). RNA hybridization was performed in a hybridization oven (Micro-4; Hybaid Ltd, UK) at 65°C for 20 hours in a hybridization solution (0.2 mM $Na_2HPO_4$, pH 7.2, 7% [vol/vol] SDS, 1% [wt/vol] BSA, and 1 mM EDTA). Gels were exposed to Kodak X-Omat AR film at −70°C with two intensifying screens. The autoradiograms were quantified using a phosphoimager (Imagequant; Molecular Dynamics, Sunnyvale, CA, USA). IGF-I mRNA levels were determined using a solution hybridization/RNase protection assay. In brief, 25 $\mu$g of total RNA was hybridized with 400,000 cpm of $^{32}$P-labeled riboprobe for 16 hours at 45°C in 75% formamine–0.4 M NaCl, followed by digestion with 40 g/L RNase T1. Protected hybrids were precipitated, denatured, and electrophoresed through 8% urea–polyacrylamide gels. Gels were exposed to Kodak X-Omat AR film as described above, and the protected band (231 bp) on the autoradiography that corresponds to IGF-I was analyzed. The autoradiograms were quantified using a phosphoimager as described above.

### Statistics

Student's $t$ test for unpaired comparisons was used. A $P$ value of less than 5% was regarded as significant. Means ± SEM are given.

### RESULTS

### Body Weight, Metabolic Parameters, and Food Consumption

At day 14, the STZ-treated mice were markedly diabetic, with blood glucose levels 4-fold higher than those of the control mice. Also, food consumption and kidney weight were increased, and liver weight was decreased in the STZ diabetic mice (Table 1).

### Serum GH, IGF-I, and IGFBP-3

No differences were observed in the basal GH levels. Following GHS stimulation, however, the serum GH levels were found to be 150 ± 35 and 62 ± 11 $\mu$g/L, respectively, in the diabetic compared with the nondiabetic animals ($P < .05$) (Figure 1). Differences in the basal serum IGF-I levels were observed between the diabetic and nondiabetic animals, being 208 ± 23 and 418 ± 22 $\mu$g/L, respectively ($P < .0001$). After stimulation, the serum IGF-I level did not change in the diabetic treated animals, but increased to 522 ± 30 $\mu$g/L in the nondiabetic animals ($P < .01$).

A representative autoradiogram of the IGFBP-3 WLB is shown in Figure 2*A*. The IGFBP-3 levels were lower in diabetic as compared to nondiabetic animals, but ipamorelin stimulation did not induce any changes neither in diabetic nor in nondiabetic animals (Figure 2*B*).

### Hepatic GHR/GHBP mRNA, IGF-I mRNA, and IGF-I Peptide

Autoradiograms of the ethidium bromide staining of the RNA samples before their transfer to the membrane and the Northern blot analysis for the GHR and GHBP mRNAs are shown in Figures 3*A* and 3*B*, respectively. Despite the fact that the basal GH levels were comparable in diabetic and nondiabetic animals, a significant lower GHR mRNA expression was found in the diabetic animals. After ipamorelin injection, the GHR mRNA level rose significantly, but only in the nondiabetic

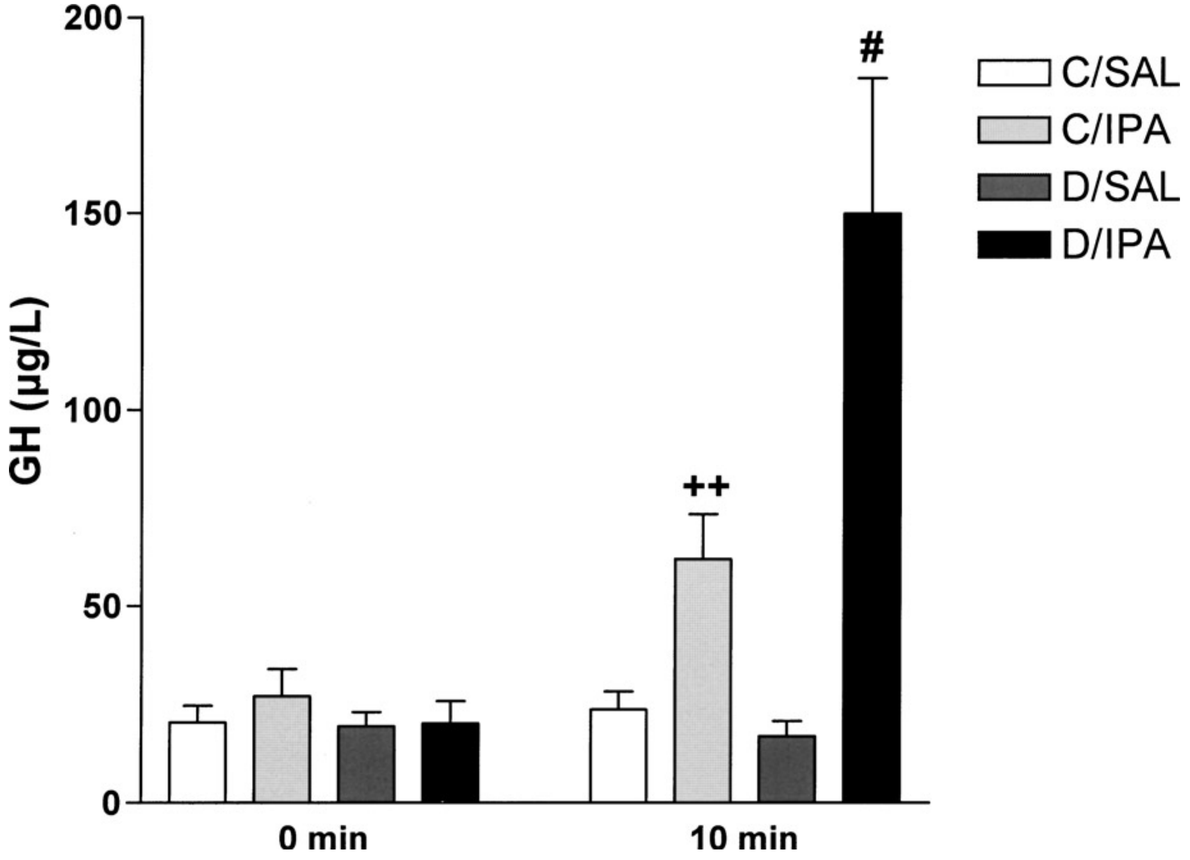

**FIGURE 1**

Serum growth hormone (GH) concentrations ($\mu$g/L) in nondiabetic mice injected with saline (C/SAL) or ipamorelin (C/IPA) and diabetic animals injected with saline (D/SAL) or ipamorelin (D/IPA) 10 minutes after injection at day 14. Values are means ± SEM, n = 7–8. $^{\#}P < .05$ compared with C/IPA; $^{++}P < .01$ compared with C/SAL. Student's *t* tests for unpaired comparisons were used.

animals. Also, GHBP mRNA levels were lower in diabetic animals, but the expression was unchanged by ipamorelin stimulation. IGF-I mRNA expression and hepatic IGF-I peptide concentrations were increased by ipamorelin stimulation in the nondiabetic animals, whereas no changes were induced in the diabetic animals. The quantitative Northern blot analysis is shown in Figure 4.

### DISCUSSION

Poorly controlled type 1 diabetes in humans is characterized by GH hypersecretion [6, 8], whereas GH secretion in diabetic rats is decreased [10, 11]. There is, on the other hand, support for the contention that the difference between experimental diabetes in rats and human diabetes, with respect to the GH/IGF axis, is restricted to GH. Accordingly, identical changes have been reported for the other elements in the axis in diabetic rats and man, including changes in circulating levels of GHBP, IGF-I, and IGFBPs; for review, see [19]. Also, disturbed hepatic and renal transcriptional changes and decreased hepatic GHR number are well-described features in experimental diabetes in rats [20–23].

The major new finding of the present study is the demonstration of GH hypersecretion in STZ diabetic mice after a GH stimulation test using the GHS ipamorelin. After GHS stimulation, a 2- to 3-fold increase in serum GH levels was evident in the diabetic mice when compared to the nondiabetic control mice. The sampling time was chosen from studies in rats and mice where the systemic GH levels were shown to peak 10 minutes after IV injection of ipamorelin [24, 25]. Ipamorelin belongs to a class of peptide compounds that have been demonstrated to possess a dual action at the pituitary as well as the hypothalamic levels. Thus, GHS provokes the release of GHRH into hypophyseal portal blood [26], and it was recently reported that passive immunization with anti-GHRH serum almost completely obliterated the GH response to GHRP-6 [27]. These data clearly demonstrate that the vast majority of the GH secretion after GHS stimulation in vivo is caused by the release of GHRH.

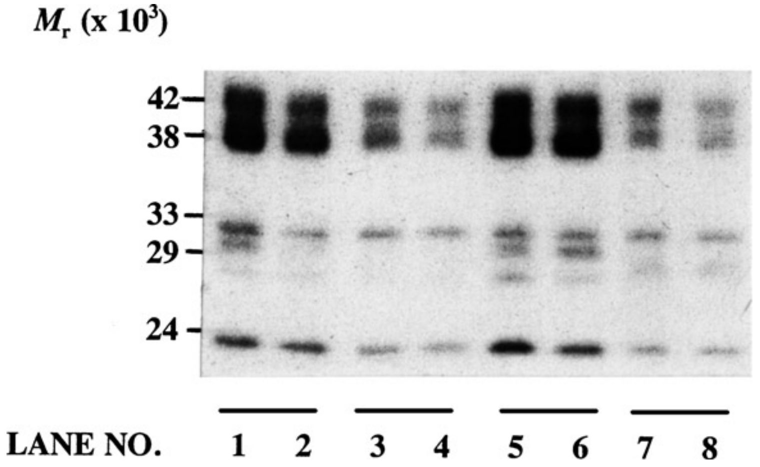

(*A*)

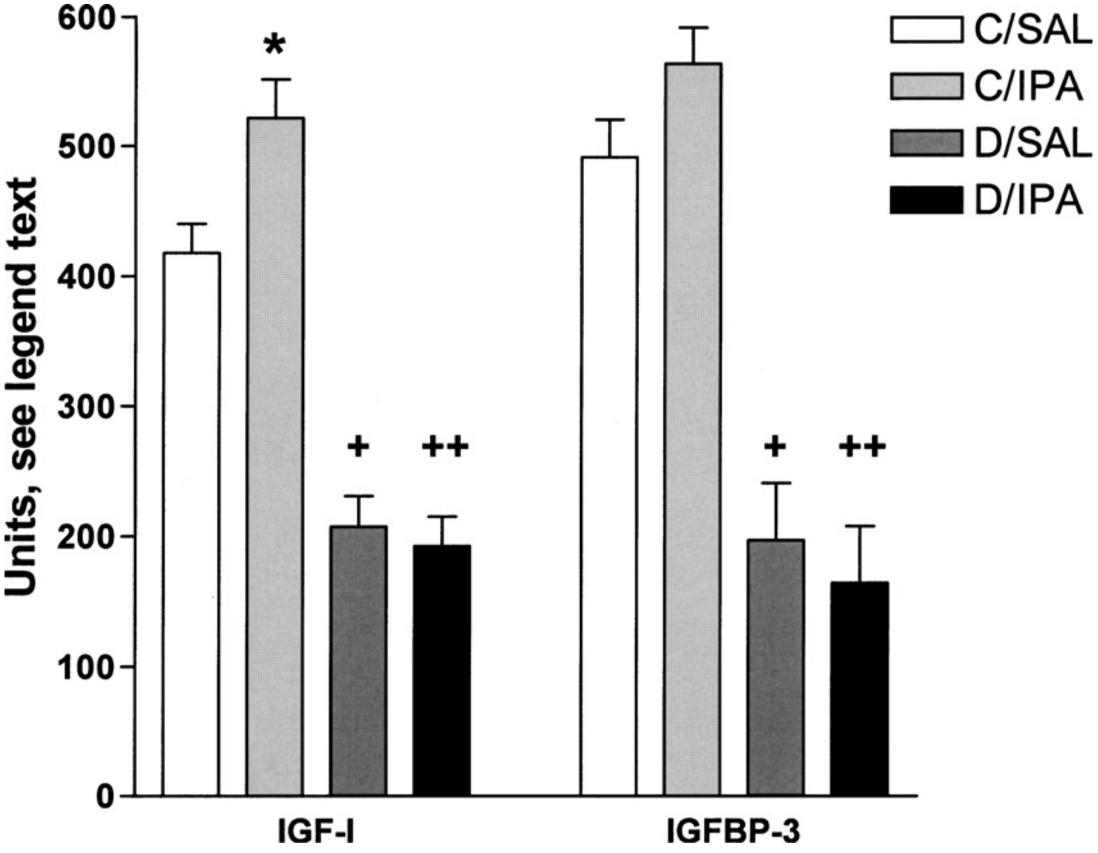

(*B*)

**FIGURE 2**

(*A*) Representative Western ligand blot (WLB) autoradiogram of serum samples from nondiabetic mice injected with saline (C/SAL) (lanes 1 and 2) or ipamorelin (C/IPA) (lanes 5 and 6) and diabetic mice injected with saline (D/SAL) (lanes 3 and 4) or ipamorelin (D/IPA) (lanes 7 and 8). Serum IGFBP-3 appears as a 38- to 42-kDa doublet band corresponding to the intact acid-stable IGF-binding subunit of IGFBP-3. $M_r$, molecular weight. (*B*) Quantitative analysis of serum insulin-like growth factor I (IGF-I) concentrations ($\mu$g/L) and IGF-binding protein 3 (IGFBP-3) levels (pixel) in nondiabetic mice injected with saline (C/SAL) or ipamorelin (C/IPA) and diabetic animals injected with saline (D/SAL) or ipamorelin (D/IPA) 10 minutes after injection at day 14. Values are means $\pm$ SEM, n = 7–8. $^{*}P < .02$ compared with C/SAL; $^{+}P < .0001$ compared with C/SAL; $^{++}P < .0001$ compared with C/IPA. Student's *t* tests for unpaired comparisons were used.

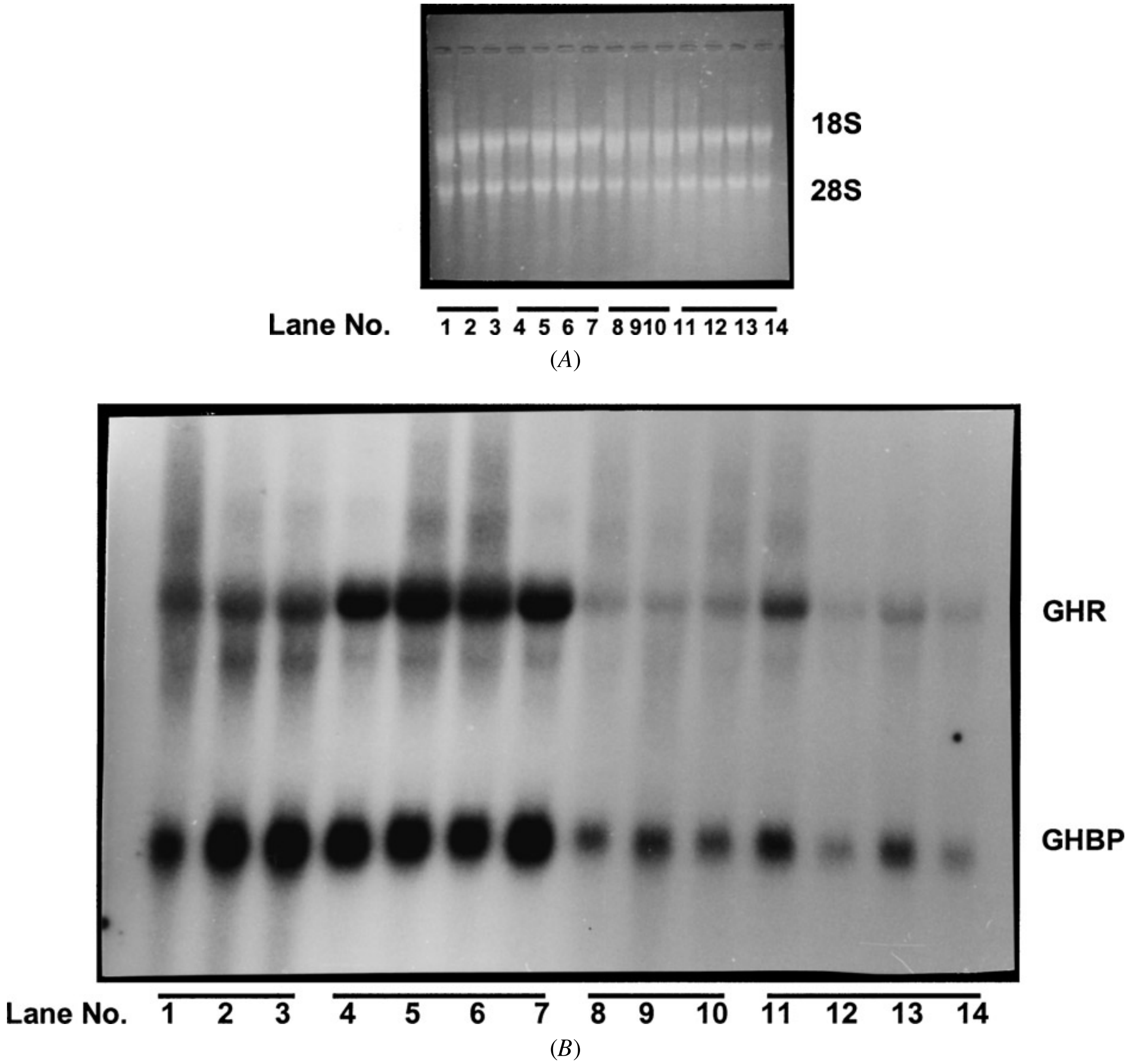

**FIGURE 3**

(*A*) Ethidium staining of the RNA samples before their transfer to the membrane. The locations of the 28S and 18S ribosomal RNA bands are depicted. (*B*) Northern blot analysis of liver total RNA (20 $\mu$g) from nondiabetic mice injected with saline (C/SAL) (lanes 1 to 3) or ipamorelin (C/IPA) (lanes 4 to 7) and diabetic mice injected with saline (D/SAL) (lanes 8 to 10) or ipamorelin (D/IPA) (lanes 11 to 14). The 4.4-kb and 1.2-kb bands corresponding to the GHR and GHBP are shown on the left.

The elevated stimulated serum GH in diabetic mice is contrary to previous reports on the usual model of animal type 1 diabetes, the STZ diabetic rat. In this animal, decreased GH levels have been described, probably due to the inhibition of GH release from the pituitary [11]. It should be noted, however, that the diabetic state in the present study and in all studies investigating GH secretion in rats have used a single high-dose STZ injection. Induction of type 1 diabetes in rats or mice can also be carried out by repeated daily low-dose STZ injections, which are less likely to give kidney toxicity and ketosis-prone diabetes. In the present study, however, the mice had a stable B-glucose without signs of ketosis. It has previously been demonstrated that a single high-dose STZ injection does not inflict kidney toxicity per se, except for the well-known kidney changes caused by the diabetic state [16]. Whether a difference in GH affection exists between the 2 STZ regimens remains to be shown.

The stimulated GH levels of the nondiabetic mice were accompanied by increases in circulating and hepatic IGF-I peptide and mRNA levels. These changes were absent in the

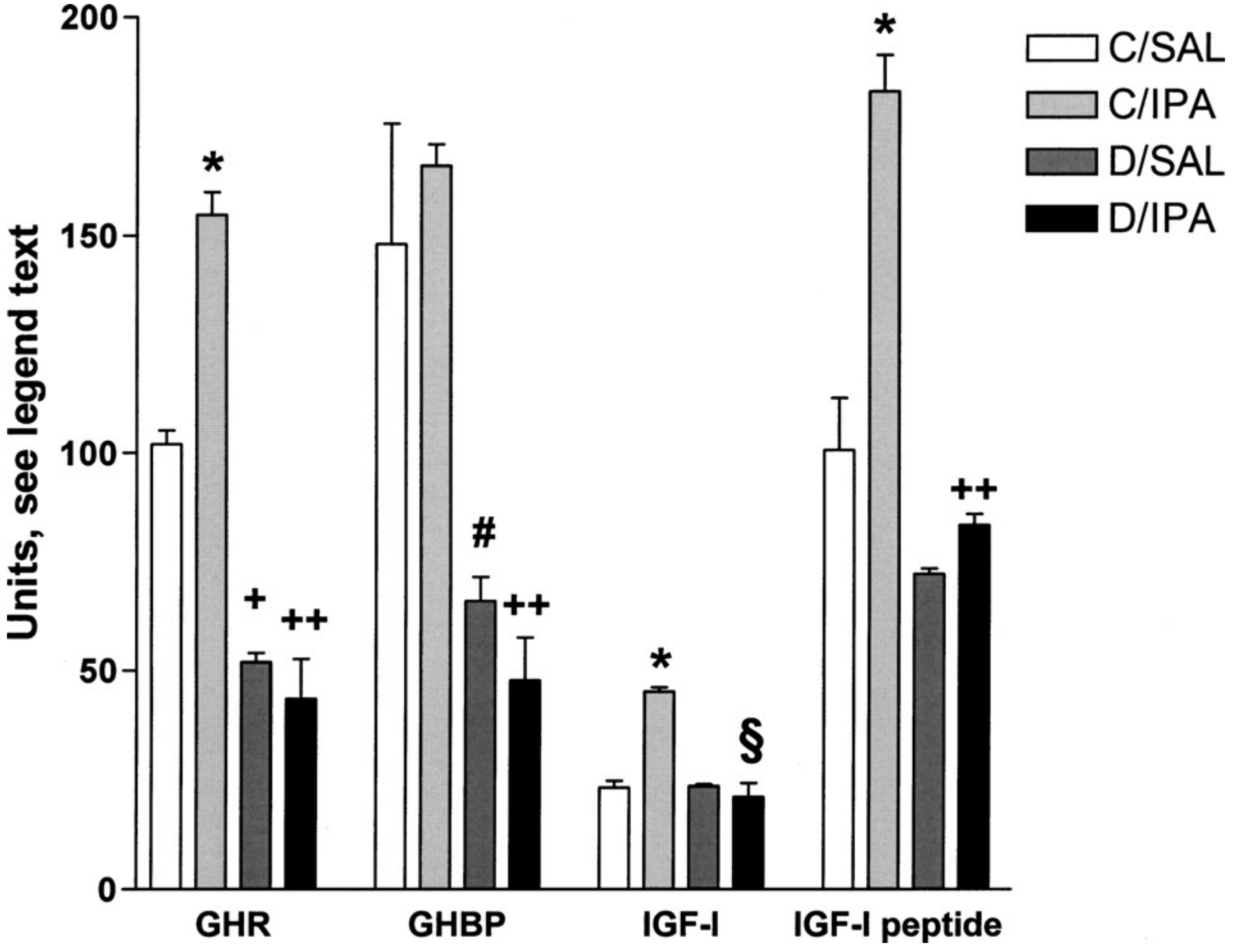

**FIGURE 4**

Quantitative analysis of hepatic GHR, GHBP, IGF-I mRNA (pixel), and hepatic IGF-I peptide (ng/g) levels in nondiabetic mice injected with saline (C/SAL) or ipamorelin (C/IPA) and diabetic animals injected with saline (D/SAL) or ipamorelin (D/IPA) 10 minutes after injection at day 14. Values are means ± SEM, n = 3–4. $^{*}P < .002$ compared with C/SAL; $^{+}P < .0002$ compared with C/SAL; $^{++}P < .0001$ compared with C/IPA; $^{\#}P < .05$ compared with C/SAL; $^{\S}P < .0005$ compared with C/IPA. Student's *t* tests for unpaired comparisons were used.

diabetic animals. Traditionally, the majority of circulating IGF-I is synthesized in the liver, mainly under the control of circulating GH, and thus in hyperglycemic diabetic subjects, serum IGF-I is reduced in spite of elevated GH. In order to explain this phenomenon, it has been hypothesized that the diabetic metabolic deterioration first decreases hepatic IGF-I formation and serum IGF-I levels, which then secondarily induces GH hypersecretion [1, 3, 5–8]. This hypothesis is in accordance with the findings of the present study, and is in agreement with results obtained in recent studies in diabetic mice [3, 16, 28]. GH receptor resistance has been suggested by previous clinical and experimental studies in type 1 diabetes. The previous findings include (a) decreased liver GHBP in diabetic states; (b) reduced plasma concentrations of GHBP in untreated spontaneously diabetic BB rats with full normalization during insulin administration [23]; (c) decreased serum GHBP in human patients with insulin-dependent diabetes mellitus (IDDM) [29]; and (d) reduced IGF-I secretion associated with low hepatic GHR levels, which also normalize during insulin treatment [30].

To our knowledge, only one study has addressed the GH profile in conscious STZ diabetic mice fitted with indwelling catheters [31]. In this study, suppression of episodic GH secretion was reported. The pituitary GH responses in vitro to different doses of GHRH, however, were found to be remarkably increased, suggesting an up-regulation of the pituitary GHRH receptor caused by a decrease in hypothalamic GHRH secretion. Also, the pituitary GH content was reduced. If the reduced GH levels in vivo were caused by decreased GHRH synthesis/release, the enhanced GH secretion following GHS stimulation in the present study is difficult to explain, because the GH release following GHS stimulation is predominantly conveyed by GHRH. One conspicuous difference between the 2 studies was found in the blood glucose levels (32 versus 24 mmol/L), suggesting that the diabetic aberration had been more severe in the study by Murao and colleagues [31]. It can be speculated that the differences in GH levels are due to differences in diabetic aberration. Of note, no differences in the basal GH levels were observed in the present study. This is contrary to the findings in nonobese diabetic (NOD) mice [28].

Recent studies have described renoprotective effects of GH antagonists in long-term diabetic transgenic mice that express the GH antagonists bGH-G119R and hGH-G120R [16, 32, 33]. Also, normalization of diabetic renal and glomerular hypertrophy, along with a reduction in the diabetes-associated increase in urine albumin excretion, have been reported in diabetic mice treated with the GH antagonist G120K-PEG [16, 34]. These observations are in agreement with the present finding of GH hypersecretion in STZ diabetic mice and that GH (and IGFs) is involved in the pathogenesis of diabetic kidney disease in experimental diabetes.

In conclusion, the present paper reports enhanced GH secretion in STZ diabetic mice when compared to nondiabetic mice following stimulation with a GH secretagogue. Consequently, the STZ diabetic mouse seems to reflect better the perturbations of the circulating GH/IGF levels as seen in human type 1 diabetes, and may be a more suitable model than the STZ diabetic rat. As only few studies on GH secretion in STZ diabetic mice have been published, more extended studies on this topic are highly warranted.

### REFERENCES

[1] Flyvbjerg, A. (1990) Growth factors and diabetic complications. *Diabetic Med.,* **7,** 387–399.

[2] Flyvbjerg, A., Froesch, ER, De Meyts, P, von zur Mûhlen, A, and Ørskov, H. (1995) International symposium on glucose metabolism and growth factors. *Metabolism,* **44,** 1–123.

[3] Flyvbjerg, A. (2000) Putative pathophysiological role of growth factors and cytokines in experimental diabetic kidney disease. *Diabetologia,* **43,** 1205–1223.

[4] Flyvbjerg, A., Ørskov, H., and Alberti, KGMM (Eds). (1993) *Growth Hormone and Insulin-Like Growth Factor I in Human and Experimental Diabetes*, pp. 1–322. New York, John Wiley & Sons.

[5] Hansen, Aa. P. (1972) Serum growth hormone patterns in juvenile diabetes. *Dan. Med. Bull.,* **19,** 1–32.

[6] Hansen, Aa. P., and Johansen, K. (1970) Diurnal patterns of blood glucose, serum free fatty acids, insulin, glucagon and growth hormone in normals and juvenile diabetics. *Diabetologia,* **6,** 27–33.

[7] Schaper, N. C. (1990) Growth hormone in type I diabetic and healthy man. *Acta Endocrinol. (Copenh.),* **122,** 1–47.

[8] Yde, H. (1969) Abnormal growth hormone response to ingestion of glucose in juvenile diabetics. *Acta Med. Scand.,* **186,** 499–504.

[9] Ndon, J. A., Giustina, A., and Wehrenberg, W. B. (1992) Hypothalamic regulation of impaired growth hormone secretion in diabetic rats. 1. Studies in streptozotocin-induced diabetic rats. *Neuroendocrinology,* **55,** 500–505.

[10] Ortiz-Caro, J., Gonzalez, C., and Jolin, T. (1984) Diurnal variations of plasma growth hormone, thyrotropin, thyroxine, and triiodothyronine in streptozotocin-diabetic and food-restricted rats. *Endocrinology,* **115,** 2227–2232.

[11] Tannenbaum, G. S. (1981) Growth hormone secretory dynamics in streptozotocin diabetes: Evidence of a role for endogenous circulating somatostatin. *Endocrinology,* **108,** 76–82.

[12] Bluet-Pajot, M. T., Durand, D., and Kordon, C. (1983) Influence of streptozotocin-induced diabetes on growth hormone secretion in the rat. *Neuroendocrinology,* **36,** 307–309.

[13] Serri, O., and Brazeau, P. (1987) Growth hormone responsiveness in vivo and in vitro to growth hormone releasing factor in the spontaneously diabetic BB Wistar rat. *Neuroendocrinology,* **46,** 162–166.

[14] Ndon, J. A., Giustina, A., and Wehrenberg, W. B. (1992) Hypothalamic regulation of impaired growth hormone secretion in diabetic rats. 2. Studies in spontaneously diabetic BB Worcester rats. *Neuroendocrinology,* **55,** 506–511.

[15] Giustina, A., and Veldhuis, J. D. (1998) Pathophysiology of the neuroregulation of growth hormone secretion in experimental animals and the human. *Endocrine Rev.,* **19,** 717–797.

[16] Flyvbjerg, A., Bennett, W. F., Rasch, R., Kopchick, J. J., and Scarlett, J. A. (1999) Inhibitory effect of a growth hormone receptor antagonist (G120K-PEG) on renal enlargement, glomerular hypertrophy, and urinary albumin excretion in experimental diabetes in mice. *Diabetes,* **48,** 377–382.

[17] Hossenlopp, P., Seurin, D., Segovia-Quinson, B., Hardouin, S., and Binoux, M. (1986) Analysis of serum insulin-like growth factor binding proteins using western blotting: Use of the method for titration of the binding proteins and competitive binding studies. *Anal Biochem.,* **154,** 138–143.

[18] Flyvbjerg, A., Kessler, U., Dorka, B., Funk, B., Ørskov, H., and Kiess, W. (1992) Transient increase in renal insulin-like growth factor binding proteins during initial kidney hypertrophy in experimental diabetes in rats. *Diabetologia,* **35,** 589–593.

[19] Flyvbjerg, A. (1997) Role of growth hormone, insulin-like growth factors (IGFs) and IGF-binding proteins in the renal complications of diabetes. *Kidney Int.,* **60,** S12–S19.

[20] Bornfeldt, K. E., Arnqvist, H. J., Enberg, B., Mathews, L. S., and Norstedt, G. (1989) Regulation of insulin-like growth factor-I and growth hormone receptor gene expression by diabetes and nutritional state in rat tissues. *J. Endocrinol.,* **122,** 651–656.

[21] Maes, M., Ketelslegers, J. M., and Underwood, L. E. (1983) Low plasma somatomedin-C in streptozotocin-induced diabetes mellitus. Correlation with changes in somatogenic and lactogenic liver binding sites. *Diabetes,* **32,** 1060–1069.

[22] Maes, M., Underwood, L. E., and Ketelslegers, J. M. (1986) Low serum somatomedin-C in insulin-dependent diabetes: Evidence for a postreceptor mechanism. *Endocrinology,* **118,** 377–382.

[23] Massa, G., Verhaeghe, J., Vanderschueren-Lodeweyckx, M., and Bouillon, R. (1993) Normalization of decreased plasma concentrations of growth hormone-binding protein by insulin treatment in spontaneously diabetic BB rats. *Horm. Metab. Res.,* **25,** 325–326.

[24] Johansen, P. B., Nowak, J., Skjaerbaek, C., Flyvbjerg, A., Andreassen, T. T., Wilken, M., and Ørskov, H. (1999) Ipamorelin, a new growth-hormone-releasing peptide, induces longitudinal bone growth in rats. *Growth Horm. IGF Res.,* **9,** 106–113.

[25] Johansen, P. B., van Neck, J. W., and Flyvbjerg, A. (2000) Effects of ipamorelin on GH secretion, GH receptor, GH binding protein, and IGF-I mRNA in mice. *Growth Horm. IGF Res.,* **10,** P128.

[26] Guillaume, V., Magnan, E., Cataldi, M., Dutour, A., Sauze, N., Renard, M., Razafindraibe, H., Conte-Devolx, B., Deghenghi, R., and Lenaerts, V. (1994) Growth hormone (GH)-releasing hormone secretion is stimulated by a new GH-releasing hexapeptide in sheep. *Endocrinology,* **135,** 1073–1076.

[27] Tannenbaum, G. S., and Bowers, C. Y. (2001) Interactions of growth hormone secretagogues and growth hormone-releasing hormone/somatostatin. *Endocrine,* **14,** 21–27.

[28] Landau, D., Segev, Y., Eshet, R., Flyvbjerg, A., and Phillip, M. (2000) Changes in the growth hormone-IGF-I axis in non-obese diabetic mice. *Int. J. Exp. Diabetes. Res.,* **1,** 9–18.

[29] Mercado, M., Molitch, M. E., and Baumann, G. (1992) Low plasma growth hormone binding protein in IDDM. *Diabetes,* **41,** 605–609.

[30] Postel-Vinay, M. C., Cohen-Tanugi, E., and Charrier, J. (1982) Growth hormone receptors in rat liver membranes: effects of fasting and refeeding, and correlation with plasma somatomedin activity. *Mol. Cell Endocrinol.,* **28,** 657–669.

[31] Murao, S., Sato, M., Tamaki, M., Niimi, M., Ishida, T., and Takahara, J. (1995) Suppression of episodic growth hormone secretion in streptozotocin-induced diabetic mice: Time-course studies on the hypothalamic pituitary axis. *Endocrinology,* **136,** 4498–4504.

[32] Chen, N. Y., Chen, W. Y., and Kopchick, J. J. (1996) A growth hormone antagonist protects mice against streptozotocin induced glomerulosclerosis even in the presence of elevated levels of glucose and glycated hemoglobin. *Endocrinology,* **137,** 5163–5165.

[33] Esposito, C., Liu, Z. H., Striker, G. E., Phillips, C., Chen, N. Y., Chen, W. Y., Kopchick, J. J., and Striker, L. J. (1996) Inhibition of diabetic nephropathy by a GH antagonist: A molecular analysis. *Kidney Int.,* **50,** 506–514.

[34] Segev, Y., Landau, D., Rasch, R., Flyvbjerg, A., and Phillip, M. (1999) Growth hormone receptor antagonism prevents early renal changes in nonobese diabetic mice. *J. Am. Soc. Nephrol.,* **10,** 2374–2381.

